# Label-free *in vivo* molecular imaging of underglycosylated mucin-1 expression in tumour cells

**DOI:** 10.1038/ncomms7719

**Published:** 2015-03-27

**Authors:** Xiaolei Song, Raag D. Airan, Dian R. Arifin, Amnon Bar-Shir, Deepak K. Kadayakkara, Guanshu Liu, Assaf A. Gilad, Peter C. M. van Zijl, Michael T. McMahon, Jeff W. M. Bulte

**Affiliations:** 1 Division of MR Research, The Russell H. Morgan Department of Radiology and Radiological Science, The Johns Hopkins University School of Medicine, Baltimore, Maryland 21205, USA; 2 Cellular Imaging Section and Vascular Biology Program, Institute for Cell Engineering, the Johns Hopkins University School of Medicine, Baltimore, Maryland 21287, USA; 3 Department of Oncology, The Johns Hopkins University School of Medicine, Baltimore, Maryland 21287, USA; 4 F.M. Kirby Research Center for Functional Brain Imaging, Kennedy Krieger Institute, Baltimore, Maryland 21205, USA; 5 Department of Biomedical Engineering, The Johns Hopkins University School of Medicine, Baltimore, Maryland 21205, USA; 6 Department of Chemical & Biomolecular Engineering, The Johns Hopkins University Whiting School of Engineering, Baltimore, Maryland 21218, USA

## Abstract

Alterations in mucin expression and glycosylation are associated with cancer development. Underglycosylated mucin-1 (uMUC1) is overexpressed in most malignant adenocarcinomas of epithelial origin (for example, colon, breast and ovarian cancer). Its counterpart MUC1 is a large polymer rich in glycans containing multiple exchangeable OH protons, which is readily detectable by chemical exchange saturation transfer (CEST) MRI. We show here that deglycosylation of MUC1 results in >75% reduction in CEST signal. Three uMUC1^+^ human malignant cancer cell lines overexpressing uMUC1 (BT20, HT29 and LS174T) show a significantly lower CEST signal compared with the benign human epithelial cell line MCF10A and the uMUC1^−^ tumour cell line U87. Furthermore, we demonstrate that *in vivo* CEST MRI is able to make a distinction between LS174T and U87 tumour cells implanted in the mouse brain. These results suggest that the mucCEST MRI signal can be used as a label-free surrogate marker to non-invasively assess mucin glycosylation and tumour malignancy.

## Abstract (web summary)

Overexpression of underglycosylated MUC1 (uMUC1) is found in most malignant adenocarcinomas of epithelial origin. Here the authors use chemical exchange saturation transfer (CEST) MRI to detect uMUC1 and to distinguish between malignant and nonmalignant tumours.

## Graphical abstract

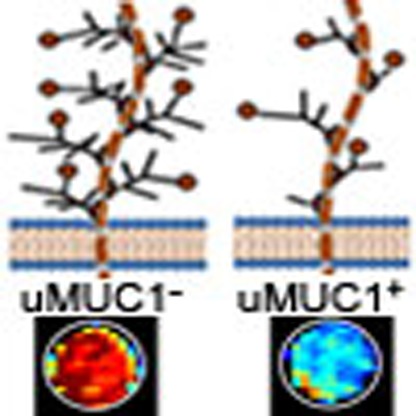

Mucins, a family of large molecular weight and heavily glycosylated proteins, constitute the mucous barrier at the epithelial surface and play an important role in cell signal transduction^1^. Alterations in mucin expression or glycosylation have long been associated with the development of cancer, as they are thought to influence cellular growth, invasion, metastasis and immune surveillance^2^,^3^. Mucin-1, one of the cell-surface-associated mucins encoded by the *MUC1* gene, is expressed aberrantly in ~900,000 of the 1.4 million tumours diagnosed each year in the United States^2^. Studies have shown that MUC1-overexpressing breast, colon and thyroid cancer cells are unresponsive to chemotherapeutic agents^4^,^5^. MUC1 is characterized by a long core protein that extends 200–500 nm beyond the cell surface and contains up to 120 tandem repeats of peptides^6^,^7^, which is rich in serines, threonines and prolines, including five potential O-linked glycosylation sites. In normal epithelial cells, MUC1 is extensively glycosylated, with >50% of its molecular mass attributable to oligosaccharide chains: the 120–225 kDa core protein mass increases to 250–500 kDa after glycosylation^8^. However, in tumour cells that develop from normal cells, MUC1 is often underglycosylated with fewer and truncated oligosaccharide side chains, identified as the tumour-associated underglycosylated MUC1 (uMUC1) antigen (Fig. 1)^6^,^9^,^10^. The reduced glycosylation of tumour cells allows exposure of a highly immunogenic core peptide epitope of the uMUC1 antigen, which has been exploited for the development of immunotherapeutic vaccines^11^,^12^,^13^,^14^ and targeted radiotheraputic drugs^15^,^16^, and is also widely used as a serum diagnostic assay to detect ovarian, breast and colon adenocarcinomas^17^,^18^,^19^.

Given its association with tumour malignancy, it is highly desirable to develop a non-invasive technique for imaging of uMUC1 overexpression. Targeted imaging agents against the uMUC1 antigen recognizing the exposed peptide sequence on the tandem repeat have been developed, including radiolabelled agents^15^,^17^,^20^ and a dual-modality probe with the near-infrared fluorescence (NIRF) dye Cy5.5 conjugated to MRI-detectable superparamagnetic iron oxide nanoparticles^21^,^22^. However, these approaches may not readily be adapted for clinical tumour staging, as drug development and approval is a lengthy and costly process. In addition, the pharmacokinetics of the probes may be such that only a small fraction of the tumour can be targeted. An imaging technique that is ‘label-free’ (that is, that does not rely on administering an exogenous agent) and can sample the entire tumour would be extremely valuable. Chemical exchange saturation transfer (CEST) magnetic resonance imaging (MRI) is a non-invasive imaging technique that can detect biological agents via frequency-selective saturation of their exchangeable protons^23^,^24^. It is highly sensitive and can amplify signals from low-concentration agents with a factor between 10^2^ and 10^6^ compared with conventional proton spectroscopy^25^. It has been used to detect both small molecules, such as glucose^26^,^27^,^28^ and glutamate^29^ and larger polymers, including glycogen^30^ and glycosaminoglycans^31^,^32^.

As mucins are natural polymers rich in glycans, we investigated whether ‘mucCEST’ imaging would be able to differentiate benign from tumour cells based on their glycosylation level. In MUC1, a single core protein contains up to 120 tandem repeats, each of which has five potential sites of O-glycosylation; a single molecule can contain up to 600 oligosaccharide side chains. Glycosylation is initiated by the addition of an N-acetylgalactosamine (GalNAc) residue to a serine or threonine, followed by the sequential addition of carbohydrate residues, such as N-acetylglucosamine (GlcNAc), and then terminated by sialic acid, fucose or galactose (Fig. 1). As each chain contains 2–10 simple sugars with 4–5 –OH protons (8–50 total –OH protons per chain), it can be calculated that 1 nM of MUC1 contains up to 30,000 nM exchangeable protons that can participate in providing contrast on CEST imaging.

We show here that uMUC1^+^ human malignant cancer cell lines exhibit a significantly lower CEST signal compared with a benign human epithelial cell line and an uMUC1^−^ tumour cell line. When uMUC1^+^ and uMUC1^−^ tumour cells were bilaterally implanted in the mouse brain, CEST MRI was able to distinguish between the two types of tumours *in vivo*. Hence, mucCEST imaging represents a novel label-free imaging technique to non-invasively probe mucin glycosylation and tumour malignancy.

## Results

### Characterization of mucin CEST signal

As the CEST signal is pH-dependent, we first examined the CEST z-spectra (Fig. 2a) and MTR_asym_ spectra (Fig. 2b) for 5 mg ml^−1^ normal (glycosylated) mucin at pH=5.8 to 7.8. Mucin showed a broad CEST spectrum from 0.5–4 p.p.m., with a peak at~1 p.p.m., which can be assigned to the numerous exchangeable −OH protons on the glycan side chains as reported previously for glucose^26^,^27^, glycogen^30^, glycosoaminoglycans^31^,^32^ and poly-sialic acid^33^. Similar to these reports, the MTR_asym_ signal between 1 and 2.5 p.p.m. increases with decreasing pH. For mucin, the peak around 2.8 p.p.m. increases below pH=6 due to the amine protons entering the slow-to-intermediate exchange regime^29^,^34^. Figure 2c shows the MTR_asym_ spectra for saturation field strengths (*B*_1_) from 1.2 to 6.0 μT for solutions at pH=7.2. A marked increase of signal occurs between 0.5 and 2 p.p.m., indicating fast exchange of hydroxyl groups. The CEST peak shifts slightly further from water as *B*_1_ increases, which is because of the increased direct water saturation moving the maximum to the left in the MTR_asym_ calculation. We then chose *B*_1_=3.6 μT for all following experiments, as it shows a comparable CEST signal at ~1.2 p.p.m. to that at the higher *B*_1_ value, but with a less broad spectrum. Figure 2d,f show the concentration dependence of CEST spectra for neutral (pH=7.2) and mildly acidic (pH=6.6) conditions; the extracellular pH is acidic for many malignant tumours^35^. The 3.6 p.p.m. peak from the backbone amides can be clearly observed in Fig. 2d. Even at the lowest concentration of 1.25 mg ml^−1^, the MTR_asym_ peaks reach >5%, which should be easily detectable. We then examined the MTR_asym_ changes as a function of concentration for three saturation frequencies (Fig. 2f). The corresponding CEST image at 1.8 p.p.m. (Fig. 2g) demonstrates strong CEST signal changes as a function of the concentration of normal glycosylated mucin.

### Effect of deglycosylation

To further prove that the CEST contrast originated from the glycosyl groups, we performed experiments on chemically deglycosylated mucin, to mimic the underglycosylated MUC1 present on malignant tumour cells. Deglycosylated mucin could be easily differentiated from normal mucin in both the *z*-spectra and MTR_asym_ spectra (Fig. 3a,b). In the *z*-spectra, there was no observable difference at the negative frequencies for the two samples, indicating that conventional magnetization transfer imaging may not able to specifically differentiate deglycosylated mucin from glycosylated normal mucin (Fig. 3a). However, in the MTR_asym_ spectra (Fig. 3b), there is a marked reduction in CEST contrast at the characterized frequency range for mucin, that is, with values of >75% reduction at 0.5–2 p.p.m. and of >50% reduction from 2 to 4 p.p.m. These large differences enabled the production of CEST images with a clear distinction of the two mucins (Fig. 3c). The residual CEST signal for deglycosylated mucin could either arise from the core proteins, or from sialic acid and GalNAc residues that were not completely cleaved off by chemical trifluoromethane sulfonic acid treatment^36^,^37^. We confirmed near-complete deglycosylation by sodium dodecyl sulfate–PAGE (SDS–PAGE) with and without periodic acid-Schiff (PAS) glycoprotein staining, as normal (native) mucin exhibited a much higher intensity of glycosyl staining for proteins >260 kDa (Fig. 3d). Furthermore, in the SDS–PAGE gel without PAS glycoprotein staining, only the deglycosylated mucin showed a protein band at a MW of ~70 kDa, whereas in the untreated mucin this was absent (Fig. 3e). Note that in Fig. 3e the region above 260 kDa also did not stain, as the coomassie blue dye does not bind well to carbohydrate moieties^38^. The absence of the band between 70 and 100 kDa in the PAS-stained gel (Fig. 3d) indicates that the main protein content (70–100 kDa) in the deglycosylated mucin is almost free of glycans, in agreement with its reduced CEST signal (Fig. 3b).

### *In vitro* imaging of encapsulated cell lines

Next, we tested whether mucCEST imaging could differentiate between human cell lines expressing different levels of uMUC1. To prevent sedimentation of cells and achieve a homogeneous suspension, five cell lines were encapsulated in alginate-PLL-alginate hydrogels at the same density (~1,000 cells per capsule, Fig. 4a). Figure 4b,c represents the average MTR_asym_ curves for the five cell lines and for empty (no cells) control capsules at two different saturation field strengths. The three tumour cell lines expressing uMUC1 (BT20, HT29 and LS174T) exhibited a significantly lower CEST contrast as compared with MCF10A cells expressing MUC1 (normally glycosylated) and U87, a uMUC1-negative cell line, at both saturation conditions, that is, from 0.7 to 3.8 p.p.m. for *B*_1_=2.4 μT and from 0.7 to 4.8 p.p.m. for *B*_1_=3.6 μT. Note that the MTR_asym_ values above 2.5–3 p.p.m. become negative (Fig. 4b), as it is normalized using the signal at the ‘negative’ frequency with respect to water, bringing more nuclear overhauser effect contributions, especially at lower *B*_1_ (refs 39, 40). CEST contrast maps showed a clearly differential MTR_asym_ contrast at 1.8 p.p.m. for both *B*_1_ conditions (Fig. 4e,f). Conventional magnetization transfer-weighted (Fig. 4d) and T2-weighted images only showed a speckled morphology of the capsules and could not differentiate MCF10A from the other three uMUC1-positive cell lines. Multiple (*n*>3) independent encapsulation experiments were repeated for each cell line, and the CEST contrast at 1.8 p.p.m. and 3.6 μT was analysed using a one-way ANOVA multi-comparison test (Supplementary Table 1 in Supplementary Information). The uMUC1-positive group showed a significant contrast difference from the uMUC1-negative group, with F_5,14_=28.22, *P*<0.0001 (Fig. 4g). Although there are subtle differences in the mucCEST spectra of the three uMUC1^+^ adenocarcinoma cell lines (BT20, HT29 and LS174T), a pair-wised comparison did not show any significant differences between them (Supplementary Table 2 and Supplementary Fig. 1 in Supplementary Information). To validate our findings, we performed immunohistological staining using an antibody detecting full-length MUC1 (Fig. 4h). Only the MCF10A cell line demonstrated normal MUC1 expression.

### *In vivo* imaging of tumour xenografts

Finally, we tested the applicability of mucCEST imaging for differentiating tumour cells *in vivo*. MCF10A, a benign human epithelial cell line, did not form tumours when implanted in the striatum of immunodeficient mice. We then compared U87, a tumour cell line without uMUC1 expression (uMUC1^−^), with malignant LS174T cells expressing uMUC1 (uMUC1^+^). The observed size of the U87 tumours was smaller than that for the LS174T in all the mice imaged (Fig. 5a), as a result from the different growth rates of the tumours. Similar to previous glycan studies, we used the average of 0.9 and 1.2 p.p.m. as the characteristic frequency for mucin to avoid overlap with the frequency ranges of amine and amide protons. The CEST contrast map (Fig. 5b) and CEST spectra (Fig. 5c) demonstrated that uMUC1^+^ LS174T cells displayed a significantly lower CEST signal compared with U87 (uMUC1^−^) cells in all three mice tested (*P*<0.05, Fig. 5d).

## Discussion

In this study, we have demonstrated that mucCEST MRI is able to differentiate between tumour cells that are expressing normal versus underglycosylated MUC1. Using extracted, purified mucins, we found that these mucopolysaccharides display a broad peak from 0.5 to 4 p.p.m., with a signal peak around ~1 p.p.m., owing to the abundance of glycan side chains. As a model for tumour cells expressing underglycosylated MUC1 (uMUC1), we deglycosylated mucin which resulted in a striking difference between the treated and untreated mucin, with the former showing a >75% reduction of CEST signal from 0.5 to 2 p.p.m. We subsequently tested our hypothesis that underglycosylated human malignant tumour cell lines (BT20, HT29 and LS174T) showed a significantly lower CEST signal compared with a benign normally glycosylated human epithelial cell line (MCF10A) and with another uMUC1-negative cell line (U87), and found this in agreement.

As a proof-of-principle to demonstrate the feasibility of *in vivo* mucCEST imaging, a uMUC1-positive cell line (LS174T) and a uMUC1-negative cell line (U87) were inoculated into the mouse brain, with, as a result, a significantly lower contrast for LS174T compared with U87 tumours. We did choose to use the homogeneous environment of brain tissue instead of an orthotopical tumour model, as there are still challenges associated with high-field small animal CEST imaging, including motion artefacts, field inhomogeneity corrections and susceptibility artefacts arising from air-tissue interfaces. Improved CEST imaging methods^41^,^42^, better-equipped clinical scanners and larger tumour volumes may allow future orthotopic imaging in patients, where longitudinal monitoring may allow for proper quantification. For instance, amide proton transfer CEST imaging has already been applied to monitor the response to neoadjuvant chemotherapy in breast cancer patients^43^. For clinical mucCEST imaging of breast adenocarcinoma, normal breast glandular tissue provides a large glycosylation contrast, which may be used as an internal baseline reference for quantification of tumour-induced mucCEST signal changes during longitudinal monitoring. Future studies will be needed to confirm the robustness of this approach. Furthermore, as chemotherapy induces a consistent reduction in uMUC-1 levels, mucCEST MRI may be further explored as a non-invasive biomarker for an assessment of therapeutic efficacy^44^,^45^. While its clinical usefulness needs to be further demonstrated, mucCEST imaging represents the first approach to differentiate in a label-free fashion between tumour cells expressing and not expressing a single specific molecule, which has been widely studied and shown to play a significant role in tumour malignancy.

## Methods

### Phantom preparation

A commercial mucin extract from porcine stomach (Sigma-Aldrich, M2378) was used to characterize the CEST properties. This crude product contains ~1% bound sialic acid, which was obtained by digestion of hog stomach with pepsin. Mucin was dissolved in 0.01 M PBS at concentrations from 1.25 to 10 mg ml^−1^, and titrated using high concentration HCl/NaOH, to various pH values ranging from 6 to 8. The solutions were placed into 1 mm glass capillaries and assembled in a holder for CEST MR imaging. The samples were kept at 37 °C during imaging.

### Deglycosylation of mucin

**Figure 1:**
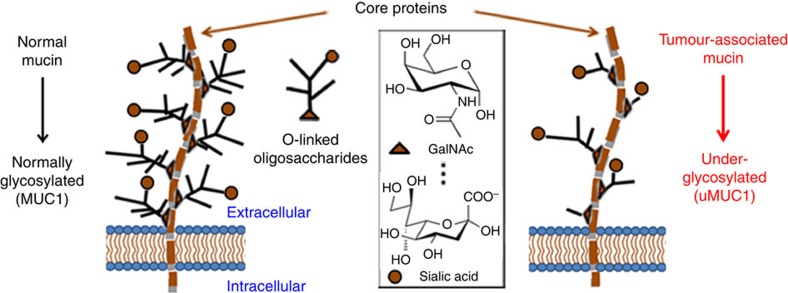
Schematic depicting the different levels of glycosylation. (Left) normal mucin (right) tumour-associated mucin. The oligosaccharide side chains consist of a variety of glycans, for example, GalNAc (triangles) that are O-linked to the core protein and the sialic acid terminal residues (circles).

**Figure 2:**
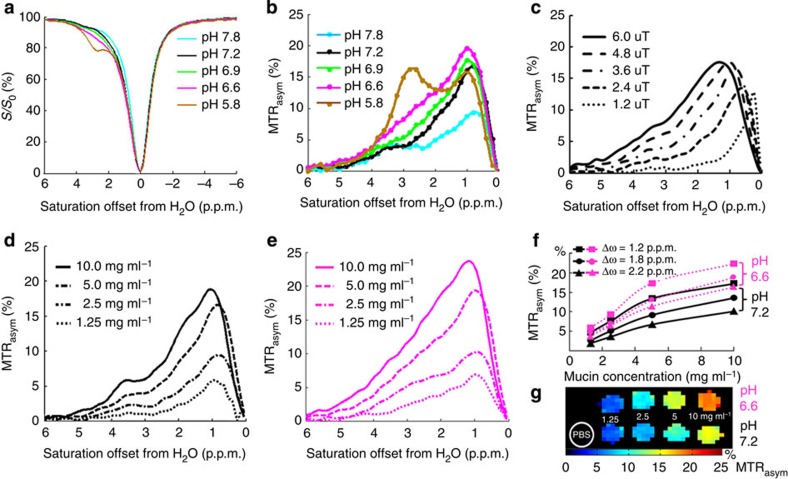
Normally glycosylated mucin exhibits a strong CEST signal. (**a**) *Z*-spectra of 5 mg ml^−1^ mucin at different pH values. (**b**) Calculated MTR_asym_ values and (**c**) dependence of MTR_asym_ on saturation power (*B*_1_) for pH=7.2. (**d**) MTR_asym_ values for different mucin concentrations at pH=7.2 and (**e**) pH=6.6. (**f**) Concentration dependence of MTR_asym_ at different offset frequencies. (**g**) Corresponding CEST image at 1.8 p.p.m.

**Figure 3:**
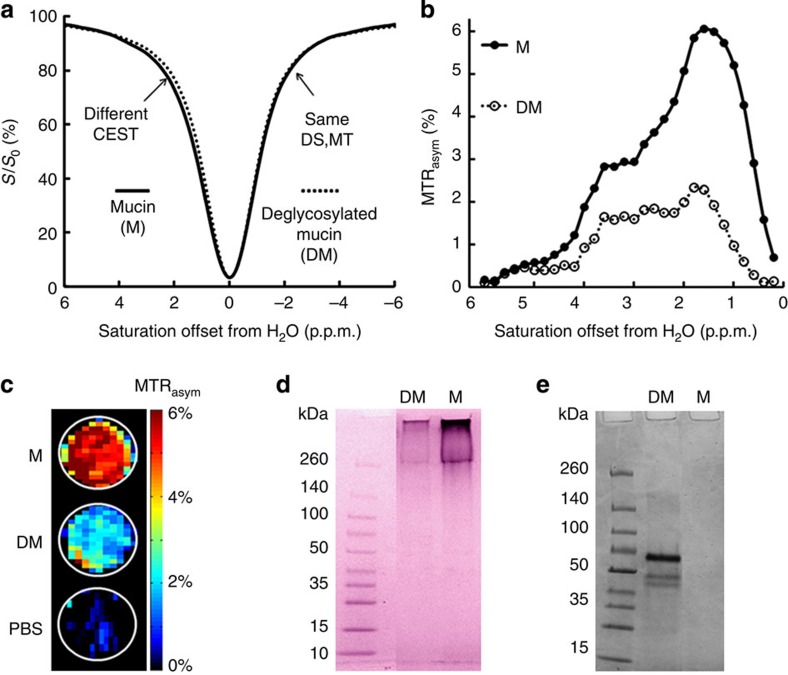
Decrease of CEST signal following deglycosylation. Experiments were performed for native (normally glycosylated) mucin (M) and deglycosylated mucin (DM). Shown are the (**a**) Z-spectra, (**b**) MTR_asym_ values and (**c**) MTR_asym_ image at 1.8 p.p.m. (**d**) PAS glycoprotein staining. (**e**) SDS–PAGE.

**Figure 4:**
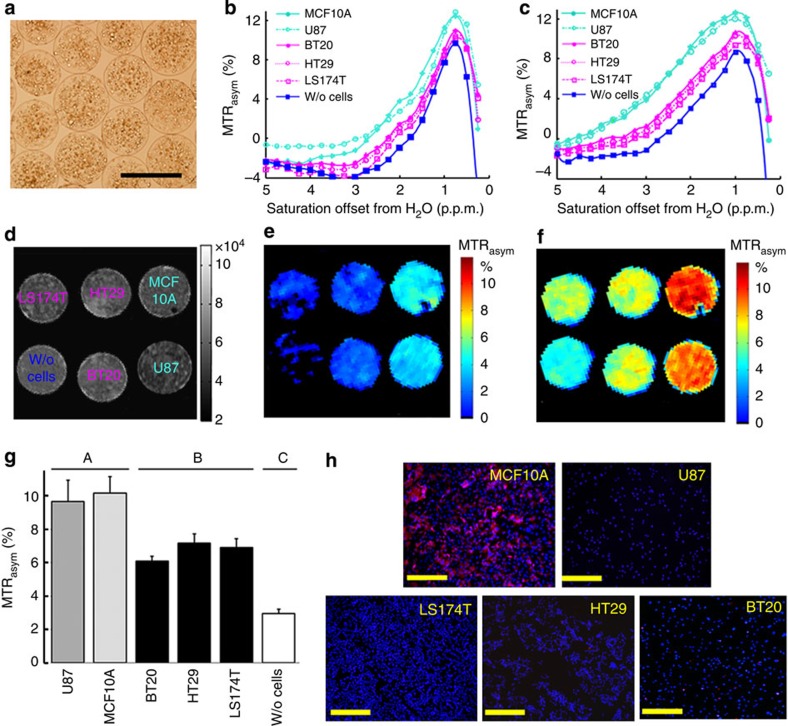
*In vitro* imaging of encapsulated cell lines. (**a**) Bright-field image (× 10) shows individual microcapsules containing MCF10A cells. Scale bar, 500 μm. (**b**) Averaged CEST spectra of the five cell lines at a saturation field strength (*B*_1_)=2.4 μT. (**c**) Averaged CEST spectra of the five cell lines at *B*_1_=3.6 μT. (**d**) MT-weighted image showing the phantom layout. (**e**) CEST contrast map at 1.8 p.p.m. with *B*_1_=2.4 μT. (**f**) CEST contrast map at 1.8 p.p.m. with *B*_1_=3.6 μT. (**g**) Statistical comparison of MTR_asym_ (1.8 p.p.m., B_1_=3.6 μT) for microcapsules with the five cell lines and without cells. Data represent means±s.e. for three independently performed experiments, analysed using one-way ANOVA (F_5,14_=28.22, *P*<0.0001) followed by a Tukey-Kramer test for multiple comparisons. Capital letters on top (A–C) indicate groups that were significantly different from all other groups tested at *P*<0.05. (**h**) Validation of MUC1 glycosylation levels using immunostaining with an antibody detecting full-length MUC1 (anti-MUC1 antibody). Red=MUC1, blue=nuclei (DAPI). Scale bar, 200 μm.

**Figure 5:**
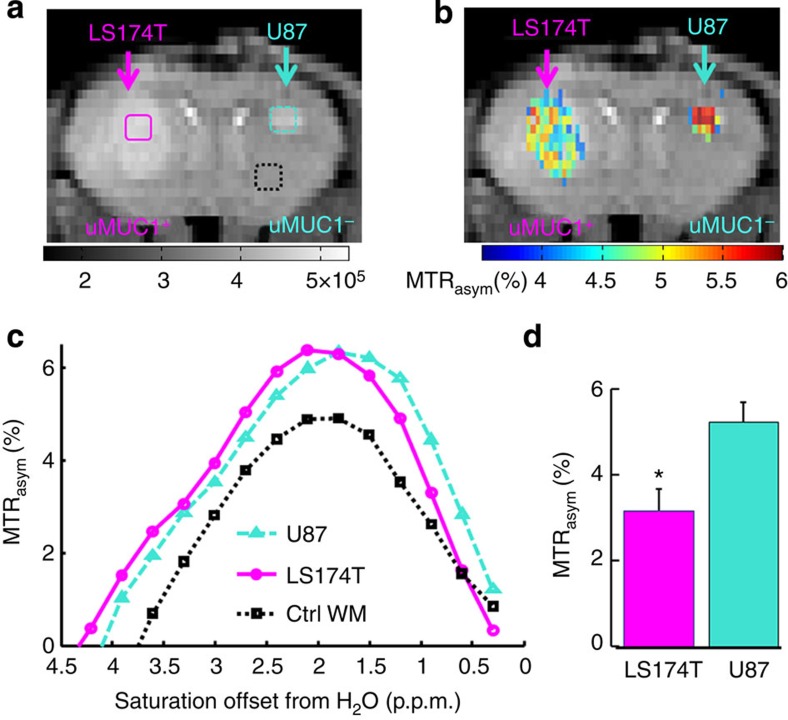
*In vivo* imaging of tumour xenografts. (**a**) T2w image, marked with regions of U87, LS174T, and control white matter (dashed square). (**b**) CEST contrast map created by averaging 1.2 and 0.9 p.p.m. superimposed onto (**a**) for *B*_1_=3.6 μT. (**c**) MTR_asym_ curves of the three ROIs marked in (**a**). (**d**) MTR_asym_ values of the two cell lines showing a significant difference (*P<0.05, Student’s *t*-test). Error bars represent s.d. (*n*=3).

We first prepared the mucins with reduced glycan chains, to mimic tumour mucins with lower glycosylation levels (Fig. 1). Owing to the complex O-linked glycosylation and polymerization of mucins, chemical deglycosylation is preferred over enzymatic methods^36^,^37^. The oligosaccharide chains on mucins (Sigma-Aldrich, M2378) were removed using anhydrous trifluoro methanesulfonic acid treatment^37^, based on the protocol of the GLYCOFREE chemical deglycosylation kit (Glyko, GKK500). After treatment, both deglycosylated and untreated mucin were dialysed against water (10 K molecular weight cutoffs) overnight, lyophilized and dissolved at ~2.0 mg ml^−1^ in PBS (pH=7.2) for imaging. To verify the glycoprotein content, the deglycosylated mucin and the untreated mucin were further analysed by using SDS–PAGE on 4–15% polyacrylamide mini-gels (Bio-Rad, Gel #456–1083S) stained with coomassie blue, with the glycosylation level confirmed by periodic acid-schiff (PAS) staining (Thermo Scientific, Pierce glycoprotein staining Kit, 24562).

### Cell culture and encapsulation

Five human carcinoma cell lines with different MUC1 glycosylation levels^21^ were used, originally obtained from ATCC (Rockville, MD). MCF10A, a benign human breast carcinoma and U87, a human glioblastoma cell line, were selected as uMUC1-negative cell lines. The three uMUC1-positive cell lines included BT20, a human breast carcinoma, and LS174T and HT29, both human colon carcinomas. U87, BT20 and LS174T cells were grown in Eagle’s minimum essential medium (MEM) with non-essential amino acids in Earle’s balanced salts solution, containing 10% fetal bovine serum (FBS) and 2% penicillin and streptomycin (all from Gibco, Grand Island, NY). HT29 cells were cultured using ATCC-formulated McCoy's 5a Medium Modified (Catalogue No. 30–2007), containing 10% FBS. The control mammary epithelial cells, MCF10A, were grown in a Mammary Epithelial Cell Growth Medium kit (Lonza, CC-3150), which contains mammary epithelial cell basal medium and growth factors, with the addition of 100 ng ml^−1^ cholera toxin. Cultures were maintained at 37 °C in a humidified atmosphere of 5% CO_2_ and 95% air. The cell media was changed every 2–3 days, and, when cells were confluent, they were 1:4 distributed to new flasks by removing cells from the surface of the culture flask gently with 0.05% trypsin EDTA and a sterile scraper.

To minimize cell sedimentation, variations in cell density and pH changes due to cell death, we encapsulated the four cell lines in alginate-PLL-alginate microcapsules at the same density of 1,000 cells per capsule. After the encapsulation, the cell-containing microcapsules were suspended in PBS, and immediately transferred to 5 mm NMR tubes for CEST imaging. The empty microcapsules without cells were also imaged as controls. To validate the MUC1 expression, immunohistological staining was performed with cells growing for 2 days in the Glass/Permanox chamber slides (Lab-Tek), using an antibody that could detect full-length MUC1 (anti-MUC1 antibody, RabMAb, from Epitomics, Burlingame CA), with red=MUC1 and blue=nuclei (DAPI).

### Animal preparation

All animal experiments were performed in accordance with the Johns Hopkins University Animal Care and Use Committee guidelines. Balb/C NOD SCID male mice (*n*=3, 6–8 weeks old) were initially anaesthetized with intraperitoneal injection of a mixture of ketamine and xylazine (0.15 ml; 62.5 and 6.25 mg kg^−1^, respectively). Mice were positioned in a stereotactic device (Stoelting Lab Standard). A small midline skin incision was made to expose the skull, and two 1 mm^2^ holes were drilled, 2 mm to left and right of bregma. A total of 1.5 × 10^5^ MCF10A, LS174T or U87 cells were bilaterally injected to the striatum of each hemisphere at a depth of 2 mm, slowly over a period of 3–4 min with the syringe removed 30 s after completion to minimize back flow. These mice were subjected to MR imaging 2–3 weeks after implantation of tumour cells. During MR imaging, mice were anaesthetized using 0.5–2% isoflurane.

### MR imaging

Imaging experiments were performed on a Bruker 11.7 T vertical bore scanner for the *in vitro* experiments and on a Bruker 9.4 T horizontal bore scanner for the *in vivo* mice experiments, both using a transmit/receive volume coil. CEST images were acquired using a continuous wave saturation pulse of 3 s as preparation, followed by a Rapid Acquisition with Relaxation Enhancement (RARE) readout sequence. The saturation field strength (*B*_1_) was varied from 1.2 to 6.0 μT for investigating the CEST properties of normal mucin phantoms, with 2.4 and 3.6 μT chosen for the cell imaging and for *in vivo* imaging. The CEST *z*-spectra were acquired by incrementing the saturation frequency every 0.2 p.p.m. from −6 to 6 p.p.m. for phantoms, and every 0.25 p.p.m. from −5 to 5 p.p.m. for cells and *in vivo*. Another set of saturation weighted images with frequency incrementing every 0.1 p.p.m. from −1 to 1 p.p.m., termed as water saturation shift reference (WASSR), was also collected for B0 mapping, using a 0.5 s saturation pulse with *B*_1_ of 0.5 μT. The other parameters are as follows: TR/ effective TE=6,000 ms/18 ms for *in vitro* and 5,000 ms/11.7 ms for *in vivo* experiments, matrix size =96 × 64, with slice thickness 1 mm. Following a pixel-by-pixel *B*_0_ correction, CEST contrast was quantified by MTR_asym_=(*S*_−Δ*ω*_–*S*_+Δ*ω*_)/*S*_0_ with *S*_+Δ*ω*_, *S*_−Δ*ω*_, *S*_0_ representing the water signal with a saturation frequency offset at +Δ*ω*, −Δ*ω* or without saturation, respectively. For the encapsulated cells, MTR_asym_=(S_−Δ*ω*_–S_+Δ*ω*_)/*S*_−Δ*ω*_ was used to increase the dynamic range.

## Additional information

**How to cite this article:** Song, X. *et al*. Label-free *in vivo* molecular imaging of underglycosylated mucin-1 expression in tumour cells. *Nat. Commun.* 6:6719 doi: 10.1038/ncomms7719 (2015).

## Supplementary Material

Supplementary Information Supplementary Figure 1 and Supplementary Tables 1-2
