# Supplementary material for: Label-free in vivo molecular imaging of underglycosylated mucin-1 expression in tumour cells
Source: Nat Commun. 2015 Mar 27;6:6719. doi: 10.1038/ncomms7719 (PMC4380237; doi:10.1038/ncomms7719)
Supplement: Supplementary Information — Supplementary Figure 1 and Supplementary Tables 1-2 [file ncomms7719-s1.pdf]

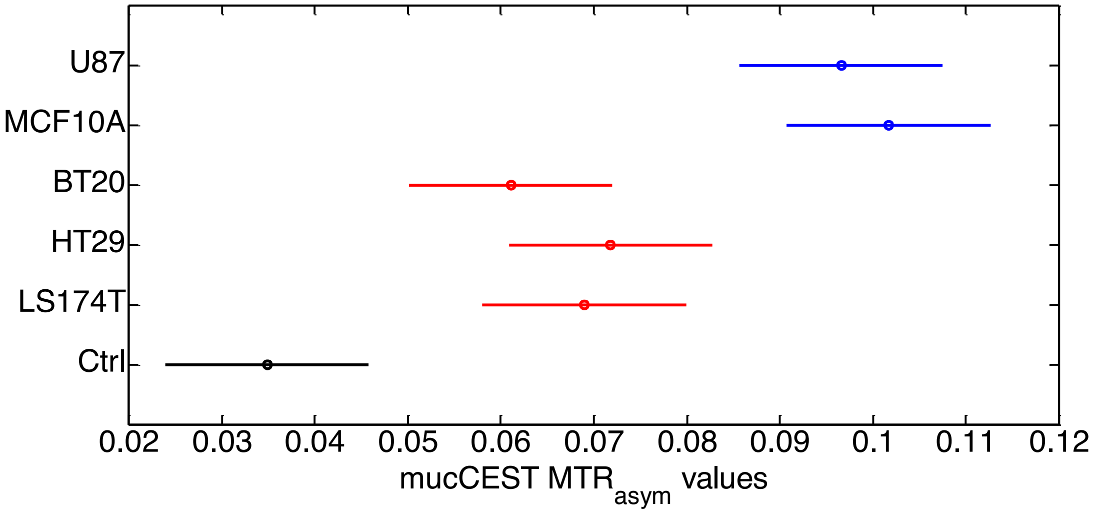

Supplementary Figure 1: Distribution of mucCEST values for the 5 cell lines used in this study. The MTR_{asym} values of the three uMUC1+ adenocarcinoma cell lines (red) are significantly different ($p<0.05$) from the two uMUC1- cell lines (blue) and capsules without cells (black).

Supplementary Table 1: *In vitro* MTR_{asym} values for the 5 different cell lines tested. Shown are the averaged MTR_{asym} values for the three independent experiments shown in Fig. 4g. Group numbers were assigned and used for statistical analysis using a one-way ANOVA multi-comparison test.

	**U87**	**MCF10A**	**BT20**	**HT29**	**LS174T**	**Control (w/o cells)**
	**Group1**	**Group2**	**Group3**	**Group4**	**Group5**	**Group6**
Expt.#1	8.2%	10.6%	6.0%	6.6%	7.0%	3.2%
Expt.#2	10.5%	10.9%	5.9%	7.6%	6.3%	2.8%
Expt.#3	10.3%	9.1%	6.4%	7.4%	7.4%	2.9%

<u>Supplementary Table 2:</u> Pair-wise comparison of MTR_{asym} values for all cell lines.
One-way ANOVA test and multi-group comparison using the MATLAB function "Anova1" and "multi-compare" based on Tukey-Kramer criteria.

			True mean difference interval (95% confidence)	
**Group#**	**Group#**	**Mean difference**	**Lower-bound**	**Upper-bound**
1*	2*	-0.5%	-2.7%	1.7%
1	3	3.6%	1.4%	5.7%
1	4	2.5%	0.3%	4.7%
1	5	2.8%	0.6%	5.0%
1	6	6.2%	4.0%	8.4%
2	3	4.1%	1.9%	6.3%
2	4	3.0%	0.8%	5.2%
2	5	3.3%	1.1%	5.5%
2	6	6.7%	4.5%	8.9%
3*	4*	-1.1%	-3.3%	1.1%
3*	5*	-0.8%	-3.0%	1.4%
3	6	2.6%	0.4%	4.8%
4*	5*	0.3%	-1.9%	2.5%
4	6	3.7%	1.5%	5.9%
5	6	3.4%	1.2%	5.6%

*Comparison of the lower-bound vs. upper-bound values for these cell groups cannot contain a significant difference, as a zero value is within the 95% confidence interval.
